# Hexavalent Chromium (Cr(VI)) Down-Regulates Acetylation of Histone H4 at Lysine 16 through Induction of Stressor Protein Nupr1

**DOI:** 10.1371/journal.pone.0157317

**Published:** 2016-06-10

**Authors:** Danqi Chen, Thomas Kluz, Lei Fang, Xiaoru Zhang, Hong Sun, Chunyuan Jin, Max Costa

**Affiliations:** 1 Department of Environmental Medicine, New York University School of Medicine, Tuxedo, New York, United States of America; 2 Department of Biochemistry and Molecular Pharmacology, New York University School of Medicine, New York, New York, United States of America; Peking University Health Science Center, CHINA

## Abstract

The environmental and occupational carcinogen Hexavalent Chromium (Cr(VI)) has been shown to cause lung cancer in humans when inhaled. In spite of a considerable research effort, the mechanisms of Cr(VI)-induced carcinogenesis remain largely unknown. Nupr1 (nuclear protein 1) is a small, highly basic, and unfolded protein with molecular weight of 8,800 daltons and is induced by a variety of stressors. Studies in animal models have suggested that Nupr1 is a key factor in the development of lung and pancreatic cancers, with little known about the underlying molecular mechanisms. Here we report that the level of Nupr1 is significantly increased in human bronchial epithelial BEAS2B cells following exposure to Cr(VI) through epigenetic mechanisms. Interestingly, Cr(VI) exposure also results in the loss of acetylation at histone H4K16, which is considered a ‘hallmark’ of human cancer. Cr(VI)-induced reduction of H4K16 acetylation appears to be caused by the induction of Nupr1, since (a) overexpression of Nupr1 decreased the levels of both H4K16 acetylation and the histone acetyltransferase MOF (male absent on the first; also known as Kat8, Myst 1), which specifically acetylates H4K16; (b) the loss of acetylation of H4K16 upon Cr(VI) exposure is greatly compromised by knockdown of Nupr1. Moreover, Nupr1-induced reduction of H4K16 acetylation correlates with the transcriptional down-regulation at several genomic loci. Notably, overexpression of Nupr1 induces anchorage-independent cell growth and knockdown of Nupr1 expression prevents Cr(VI)-induced cell transformation. We propose that Cr(VI) induces Nupr1 and rapidly perturbs gene expression by downregulating H4K16 acetylation, thereby contributing to Cr(VI)-induced carcinogenesis.

## Introduction

Chromate is a well-established carcinogen that is a contaminant at half of the toxic waste sites in the United States [1]. Cr(VI) compounds induce human respiratory cancers and increase the risk of other types of human cancers [2]. Cr(VI) has been shown to induce lung cancers by inhalation and gastrointestinal (GI) cancer by ingestion [3]. We have shown that ingestion of Cr(VI) at 0.5 to 10 ppm in the drinking water greatly enhances UV-induced skin cancer in hairless mice [2]. Cr(VI) is one of the few carcinogenic metals that directly reacts with DNA, forming adducts, and inducing mutations [4, 5]. There are a number of studies demonstrating that Cr(VI) carcinogenesis involves gene silencing and other epigenetic effects [6, 7]. Cr(VI) has been shown to prevent the expression of inducible genes in cells by crosslinking a histone deacetylase to inducible promoters [8]. The presence of this deacetylating enzyme, which removes acetyl groups from lysines in histone tails, keeps the nucleosome condensed, thereby preventing transcription factors from binding and activating gene expression [8]. In order for cells to survive chronic Cr(VI) treatment, they must adapt and evade apoptosis. The loss of apoptotic activity is often accompanied by a loss of mismatch repair, since the two processes are tightly linked. Consistent with the latter, chronic exposure of cells to Cr(VI) or tumors induced by this agent in humans are often missing mismatch DNA repair capacity [9–11]. We have shown that Cr(VI) exposure leads to silencing of MLH1, a component of mismatch repair, via a decreased mRNA expression resulting from enhanced H3K9 dimethylation of its promoter [6]. In human lung cancers induced by Dr(VI) exposure, silencing of *MLH1* as well as tumor suppressor *p16* was correlated with DNA methylation of their promoters [9, 12]. In spite of considerable research effort, the epigenetic mechanisms of Cr(VI)-induced carcinogenesis remain largely unknown.

The Nupr1gene was cloned almost 20 years ago as being activated in pancreatic acinar cells during the acute phase of pancreatitis [13, 14]. Nupr1 mRNA is strongly induced by a variety of stressors such as lipopolysaccharides [15], CCl_4_ [16], starvation [17], cell cycle arrest and many others [13]. Overexpression of Nupr1 has been implicated in a number of cancers. For example, non-small cell lung cancer (NSCLC) tissue samples showed up-regulation of Nupr1 as compared with peritumoral lung tissues. Downregulation of Nupr1 expression significantly inhibited non-small cell lung cancer H1299 cell proliferation and colony formation *in vitro* [18]. Importantly, silencing of Nupr1 by tail vein injection of lentivirus encoded shRNA against Nupr1 *in vivo* suppressed growth of human lung cancer xenograft [18], suggesting that Nupr1 plays critical roles in lung cancer development. In addition, Nupr1 might have impact on metastasis of cancers. Nupr1 is required for metastasis of breast cancer [19]. Nupr1 was also found over-expressed in thyroid neoplasm and its expression level was directly linked to lymph node metastasis in medullary thyroid carcinoma [20]. Nupr1 enhances the expression of at least two major epithelial-mesenchymal transition (EMT)-related genes, namely MMP9 and MMP13 metalloproteases [21]. Downregulation of H4K16ac is likely a mechanism whereby Nupr1 promotes cancer development. Recent study has demonstrated that Nupr1 overexpression inhibits acetylation of lysine 16 of histone H4 (H4K16ac) [22] and the histone acetyltransferase MOF (Kat8, Myst 1), which specifically acetylates H4K16 [23]. The loss of H4K16ac and MOF correlate with increased genome instability, which is considered an important step in cancer development [24–26]. The loss of H4K16ac is found in a number of tumors, including lung cancer and considered as a ‘hallmark’ of human cancer [27–29].

In this study, we investigate the role of Nupr1 in Cr(VI)-induced carcinogenesis. We find that Cr(VI) exposure leads to increase in the level of Nupr1 in human bronchial epithelial BEAS2B cells and the loss of H4K16ac. Cr(VI)-induced reduction of H4K16ac appears to be caused by the induction of Nupr1, since overexpression of Nupr1 decreases the levels of H4K16ac, while knockdown of Nupr1 by siRNA greatly compromised the loss of H4K16ac following Cr(VI) exposure. Importantly, overexpression of Nupr1 induces anchorage-independent cell growth and knockdown of Nupr1 expression prevents Cr(VI)-induced cell transformation. Together, downregulation of H4K16 acetylation through inducing Nupr1 expression might represent a new mechanism for Cr(VI) carcinogenesis.

## Materials and Methods

### Cell Culture and Treatment

The immortalized human bronchial epithelial cell line BEAS2B was originally purchased from ATCC and authenticated by short tandem repeats (STR) analysis. The BEAS2B cells were maintained in DMEM supplemented with 10% FBS, 100 U/ml penicillin, and 100 μg/ml streptomycin. Cells were incubated at 37°C in a humidified atmosphere of 5% carbon dioxide. Cells were seeded at 3 x 10^6^ in 10-cm-diameter tissue-culture dishes with growth medium. For acute chromium exposure, cells were treated with potassium chromate (K_2_CrO_4_, obtained from J.T. Baker Chemical Co.) with doses ranging from 0 to 10 μM for 24 hours. For chronic Cr exposure, cells were treated with 0 to 0.5 μM Cr for 1 to 2 weeks.

### Cell Transfection

Cell transfections were carried out using Lipofectamine 2000 (Invitrogen) according to the manufacturer’s instructions. The control siRNA or Nupr1 siRNA (Sigma) was transfected into BEAS2B cells. For Nupr1 overexpression, the pCMV-Nupr1 expressing vector (RC222237, Origene) was transfected into BEAS2B cells. pcDNA3.1-empty vector, which contains CMV promoter, was used as the control.

### Cell Lysates, Acid Extraction, and Western Blot

After treatment, cells were washed with ice-cold PBS and collected by centrifugation. Whole cell lysates were prepared by using whole cell lysis buffer (50 mM Tris-HCl, pH 7.4, 1% Triton X-100, 0.5% sodium deoxycholate, 0.1% SDS, 500 mM NaCl, 10 mM MgCl_2_, 10 mM sodium butyrate, and protease inhibitors), incubated on ice for 60 min, and centrifuged at 11,000 × g for 10 min. The supernatant was transferred to a new tube. For soft agar transformed clones, cells were resuspended in acid extraction buffer (10 mm HEPES, pH 7.5, 1.5 mm MgCl_2_, 10 mm KCl, 0.2 N HCl, 0.5 mm DTT, 10 mm sodium butyrate, and protease inhibitors), incubated on ice for 60 min, and centrifuged at 11,000 × g for 10 min. The supernatant was transferred to a new tube and neutralized with one-fifth volume of 1.5 M Tris-HCl buffer (pH 8.8). Fifty micrograms of total protein was separated on a 14% polyacrylamide gel by electrophoresis and transferred to a polyvinylidene difluoride (PVDF) membrane (Bio-Rad). The membrane was cut into different parts according to the predicted size of the target proteins. Non-specific binding sites were blocked by incubation in Tris-buffered saline (TBS) containing 0.1% Tween-20 and 5% dry non-fat milk. The membranes were immunoblotted with primary antibodies (anti-acetyl-histone H4 Lys-16 1:1000 dilution, sc8662, from Santa Cruz; anti-Nupr1, 1:1000 dilution, ab87454 and anti-tubulin, 1:1000 dilution, ab7792, from Abcam; and MOF, 1:1000 dilution, Bethyl A300-992A, from Bethyl) overnight at 4°C, followed by incubation with a peroxidase-conjugated second antibody (goat anti-rabbit IgG for H4K16Ac and MOF, goat anti-mouse IgG for Nupr1 and Tubulin). The reagent for enhanced chemiluminescence (Bio-Rad) was used for detection and developed by X-ray film.

### DNA Extraction, Bisulfite Modification and Methylation-Specific PCR (MSP)

DNA was extracted from cells using QIAamp DNA Mini Kit (Qiagen) following the manufacturer’s protocol. DNA from each sample was treated with sodium bisulfite using the EpiTect Bisulfite Kit (Qiagen) according to the manufacturer's protocol.

The methylation status of the promoter of the Nupr1 gene was determined by MSP as described by Herman *et al*. [30]. The primer sequences for detecting the methylated Nupr1 promoter were 5′-TAGGAAAGTAGAGATAGATAAAGCGT-3’ (forward) and 5′-ATTCATCCAAACTAAAATCCTCGT-3’ (reverse), and the primer sequence for the unmethylated reaction were 5’-TTAGGAAAGTAGAGATAGATAAAGTGT-3’ (forward) and 5’-ATTCATCCAAACTAAAATCCTCATC-3’ (reverse), giving an amplification product about 150 bp. MSP amplification for the Nupr1 gene was carried out in a final volume of 25 μl, 2.5 μl 10X standard Taq reaction buffer, 0.5 μl primers, 0.5 μl 10 mM dNTP_S_, 2 μl DNA template, 0.25 μl Taq DNA polymerase, and 18.75 μl ddH2O. Amplification was performed in a MyCycler Thermal Cycler machine (Bio-Rad). The PCR conditions for MSP were as follows: hot start at 95°C for 10 min; then 35 cycles: 30s at 95°C for denaturation, 45s at 61°C for annealing, 45s at 72°C for elongation followed by 5 min at 72°C for extension. Twenty microliter of each PCR reaction was loaded onto a 2% agarose gel, and visualized by ethidium bromide staining.

### RNA Extraction and Real Time Quantitative PCR

The RNA was extracted using the TRIzol reagent (Invitrogen), according to the manufacturer’s protocol. The quantity and purity of the RNA prepared from each sample were determined by UV absorbance spectroscopy. The reverse transcription reaction was performed using SuperScript III First-Strand Synthesis System (Invitrogen) with 1 μg of RNA in a final volume of 20 μl. After incubation at 50°C for 50 min, the reverse transcription reaction was terminated by heating at 85°C for 5 min. Quantitative real time PCR analysis was performed using Power SYBR Green PCR Master Mix (Applied Biosystems) on the ABI PRISM 7900HT system. All PCRs were performed in triplicate. Relative gene expression levels were normalized to Tubulin expression. The results were presented as–fold change to the level expressed in control cells. The following primers were used: Nupr1, 5′- CTGGCCCATTCCTACCTCG-3′ (forward) and 5′- TCTCTTGGTGCGACCTTTC-3′ (reverse); MOF, 5'-GGC TGGACGAGTGGGTAGACAA-3' (forward) and 5'-TGGTGA TCGCCTCATGCTCCTT-3' (reverse); IAP (inhibitor of apoptosis), 5′-TGGTTTCCAAGGTGTGAGTACTTG-3′ (forward) and 5′-GGGCTGTCTGATGTGGATAGC-3′ (reverse); D4Z4, 5′-CTCAGCGAGGAAGAATACCG -3′ (forward) and 5′- ACCGGGCCTAGACCTAGAAG-3′ (reverse); TRIM42S (tripartite motif containing 42), 5′-AGTTTCCACCAACATACCAGC -3′ (forward) and 5′-TCCCAGGACTCTTGATGCCT -3′ (reverse); and Tubulin, 5′-CGGCTGAATGACAGGTATCCTAAG -3′ (forward) and 5′-CTCGTCCTGGTTGGGAAACA -3′ (reverse).

### Nucleosome Preparation and ChIP

Mono- and dinucleosomes were isolated by micrococcal nuclease (MNase) digestion and sucrose gradient purification and subjected to ChIP analysis with H4K16ac antibody, as we described previously [31]. The following primers were used: IAP, 5′-CCGCTGGAGTTCCCCTAAG-3′ (forward) and 5′-CGCACTCCTCCCAGTGGTT-3′ (reverse). The other primers used are the same as listed for RT-qPCR.

### ChIP and PCR Analyses

Approximately 1.5 × 10^7^ to 2 × 10^7^ cells were incubated for 10 min at room temperature with 1% formaldehyde. After cross-linking, the reaction was stopped with 0.125 M glycine for 10 min at room temperature. After being rinsed twice with ice-cold 1× PBS, cells were scraped from the dishes, pelleted by centrifugation, resuspended in cell lysis buffer (50 mM Tris-HCl [pH 8.0], 10 mM EDTA, 1% sodium dodecyl sulfate, and protease inhibitor cocktail). Chromatin was sheared to a size range of 200 to 400 bp by sonication in an ice-water bath with 30 times (30s pulse and 30s rest) in Bioruptor (Diagenode). After centrifugation to remove cell debris, chromatin was precleared for 30 min at 4°C with 50% gel slurry of protein A-agarose beads saturated with bovine serum albumin (Upstate). Then cell lysates were subjected to overnight incubation at 4°C with 2 μg of H3K9&K14ac antibody (Upstate 06–599), or non-specific IgG (IgG rabbit Upstate 12–370), and 50 μl of 50% beads slurry of Dynabeads protein A (Life Technologies), followed by washes: (1) twice with RIPA buffer (0.1% SDS, 1% Triton-X-100, 20 mM Tris pH8.0, 150 mM NaCl, 0.1% sodium Deoxycholate); (2) twice with RIPA buffer containing 0.3 M NaCl; (3) twice with 0.25 M LiCl, 0.5% NP-40, 0.5% sodium Deoxycholate; (4) once with TE pH8.0 containing 0.2% Triton-X-100; and (5) once with TE pH8.0. Immunoprecipitated chromatin was de-crosslinked overnight at 65°C with 1 mg/ml ProteinaseK, 1% SDS. For total DNA input, 40 μg were similarly de-crosslinked. DNA fragments were purified by phenol/chloroform extraction and amplified by real-time quantitative PCR in 7900 Fast Sequence Detection System (Applied Biosystems).

### Sodium Butyrate and 5-Aza-2′-deoxycytidine Treatment

To test whether the Nupr1 mRNA expression level is regulated via epigenetic mechanisms, we treated BEAS2B cells with an inhibitor of histone acetylation (sodium butyrate) or DNA methylation (5-aza-2′-deoxycytidine). The BEAS2B cells were treated with 5 mM sodium butyrate for 24 h or with 10 μM 5-aza-2′-deoxycytidine for a total of 48 h with a medium change after 24 h, respectively. The cells were collected, and total RNA was extracted for quantitative real time PCR (RT-qPCR) to monitor changes in the Nupr1 mRNA level.

### Soft-agar Assays

After transfection and Cr treatment, cells were plated onto each well of a 6-well plate at a density of 5000 cells/well in growth medium containing 10% FBS and 0.35% low melting point agarose (Sigma) on a base layer of 0.5% low melting point agarose, and cultured at 37°C incubator with 5% CO_2_ for 3 weeks. The colonies were stained with INT/BCIP (Roche) and photographed. The number of colonies larger than 50 μm was counted. The experiments were carried out independently three times. The results were shown as–fold change compared with the control group.

### Statistical Analysis

Gel intensities were quantified using ImageJ image processing software (National Institutes of Health). Relevant results are represented as mean ± S.D. (error bars). Significance was assessed by Student's t test. *p* < 0.05 was considered statistically significant. The number of times that each experiment was repeated is indicated in the figure legends.

## Results

### Cr(VI) upregulates expression of Nupr1

**Fig 1:**
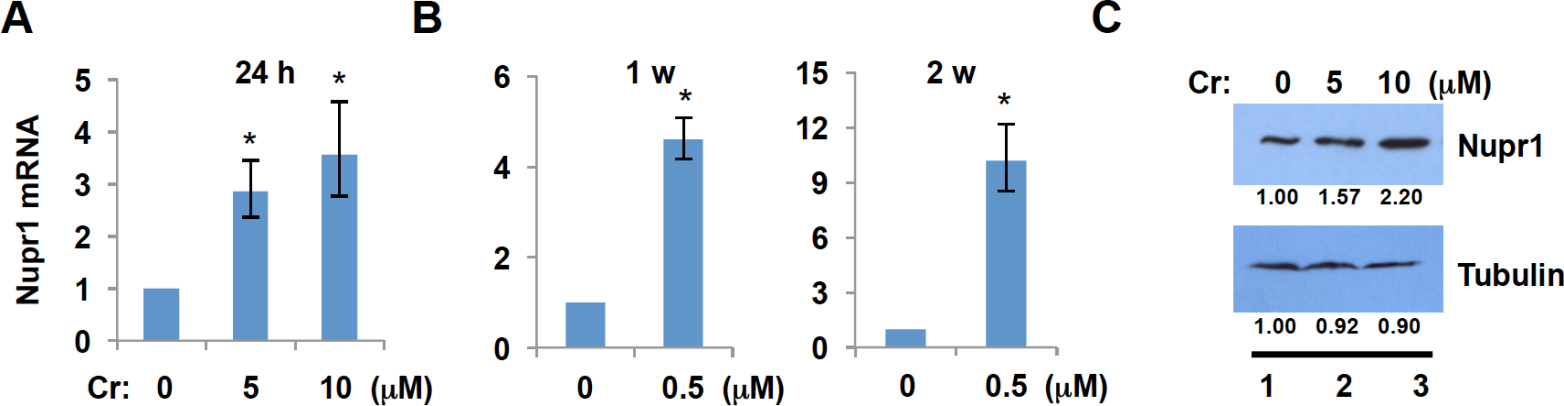
Cr(VI) induces expression of Nupr1. (A) Acute chromium exposure induces Nupr1 expression. Total RNA was extracted from BEAS2B cells after a 24 h treatment with 0, 5 or 10 μM chromium. Nupr1 mRNA levels were measured by RT-qPCR. The relative gene expression level was normalized to gamma-tubulin and is presented as fold change compared to the control group. The data shown are the mean ± S.D. from qPCRs performed in triplicate. *, *p*<0.01. (B) Chronic chromium exposure induces Nupr1 expression. After treating BEAS2B cells with 0.5 μM chromium for 1 week or 2 week, the cells were subjected to RT-qPCR using primers for Nupr1. gamma-tubulin was used as an internal control. (C) After Cr(VI) exposure, BEAS2B cells were lysed with RIPA buffer, and whole-cell lysate was run on 14% SDS acrylamide gels. Nupr1 protein levels were then measured with anti-Nupr1 antibody. The band intensities were quantified using ImageJ software.

Nupr1 is induced by a variety of stressors and the induction of Nupr1 is required for the development of lung and pancreatic cancers in animal models [18, 32]. Cr(VI) compounds are strong oxidants that induce oxidative stress in tissue culture systems and cause formation of Cr(III) adducts that mediate DNA-DNA and DNA-protein crosslinks [2, 33, 34]. We thus investigated whether Nupr1 is induced by Cr(VI) exposure and studied what roles Nupr1 have in Cr(VI)-induced carcinogenesis. We used human bronchial epithelial cell line BEAS2B in our studies, since Cr(VI) has been shown to cause lung cancer [34, 35]. We treated BEAS2B cells with 0, 5, and 10 μM Cr(VI) for 24 hours and measured the levels of Nupr1 transcripts by RT-qPCR. The levels of mRNA for Nupr1 were increased about 3- and 4-fold, respectively, by Cr(VI) exposure in a concentration dependent manner (Fig 1A). To mimic environmentally relevant doses of exposure and identify gene expression alterations, we exposed BEAS2B cells to a low-doses of Cr(VI) (0.5 μM) for up to two weeks. We observed a striking increase of Nupr1 mRNA by chronic, low-dose exposure to Cr(VI) (Fig 1B). Moreover, Western blot analysis revealed that Nupr1 protein levels were also upregulated in BEAS2B cells treated with 5 or 10 μM Cr(VI) for 24 hours (Fig 1C) and the effect was concentration dependent. These results indicate that stressor protein Nupr1 was induced by Cr(VI) exposure.

### Epigenetic induction of Nupr1 by Cr(VI)

**Fig 2:**
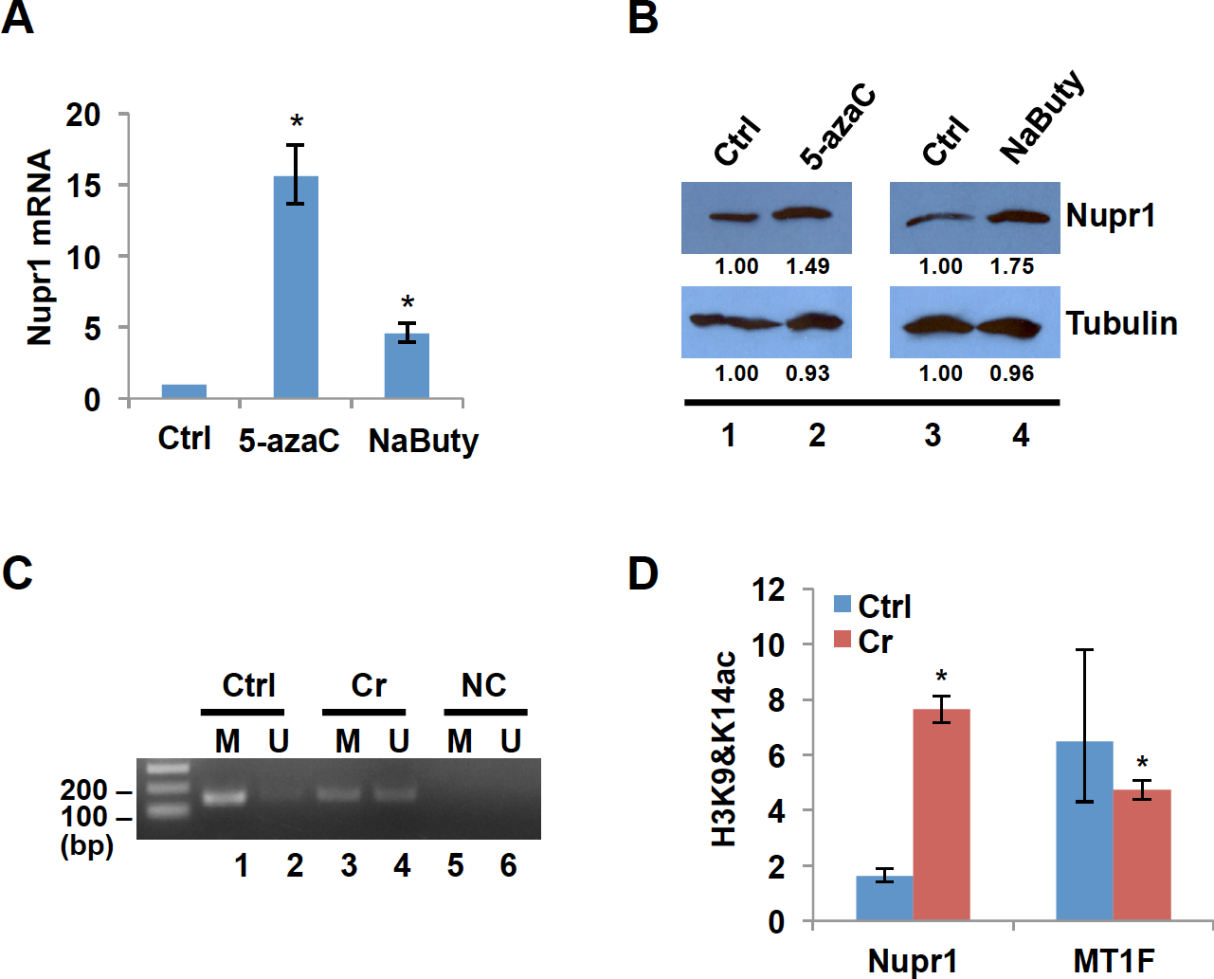
Nupr1 is induced by Cr(VI) through changes in promoter of DNA methylation and histone acetylation. (A) BEAS2B cells were treated with or without 10 μM 5-aza-2′-deoxycytidine (5-azaC) for 48 h or 5 mM sodium butyrate for 24 h. Total RNA was extracted, and Nupr1 expression was measured by RT-qPCR. The relative gene expression level was normalized to gamma-tubulin expression and is presented as fold change compared to the control group. The data shown are the mean ± S.D. from qPCRs performed in triplicate. *, *p*<0.01. (B) Total cell lysates were prepared from BEAS2B cells treated with or without 5-azaC or sodium butyrate and subjected to Western blot analysis using antibodies against Nupr1 or Tubulin. The band intensities were quantified using ImageJ software. (C) MSP analysis of Nupr1 promoter. The DNA prepared from BEAS2B cells treated with or without Cr(VI) was treated with bisulfite to convert cytosine to uracil but leave 5-methylcytosine unaffected. Primers utilized for PCR amplification were designated as methylated (M) or unmethylated (U). Amplification products were separated on 2% agarose gel and visualized by ethidium bromide staining. NC: negative control—H_2_O was used as PCR template. (D) Histone H3K9&14ac in the promoter region of Nupr1. ChIP was carried out with antibodies to H3K9&14ac. Enrichment is shown relative to the genomic abundance measured in the starting chromatin preparation (Input). Unrelated MT1 gene locus was used as a negative control. The data shown are the mean ± S.D. from qPCRs performed in triplicate. *, *p*<0.01.

Nupr1 mRNA is increased following Cr(VI) exposure (Fig 1). Veerla et al. reported that low NUPR1 expression was associated with promoter DNA methylation [36]. We and others have reported that Cr(VI) can alter gene expression by reprogramming the epigenetic status of affected genes. When we exposed human lung A549 cells to Cr(VI), we observed a specific increase in global levels of H3K4me3 (an active mark). Moreover, Cr(VI) exposure induced global hypomethylation in A549 cells [37]. Another study has demonstrated hypomethylation of the 45S rRNA gene in germ cells after Cr exposure in mice [38]. Thus, we hypothesized that Cr(VI) may regulate Nupr1 expression by modulating status of histone modifications and/or DNA methylation at its promoter region. To test whether Nupr1 expression is epigenetically regulated, we treated BEAS2B cells with sodium butyrate or 5-aza-2′-deoxycytidine (5-azaC), inhibitors of histone deacetylase and DNA methyltransferase respectively, and examined the changes in Nupr1 expression before and after the treatment. RT-qPCR and Western blot analyses respectively showed that both mRNA and protein levels of Nupr1 were increased by treatment of cells with 5-azaC or sodium butyrate (Fig 2A and 2B). The results suggest that epigenetic mechanisms such as changes in histone acetylation and DNA methylation are involved in regulation of Nupr1 expression.

**Fig 3:**
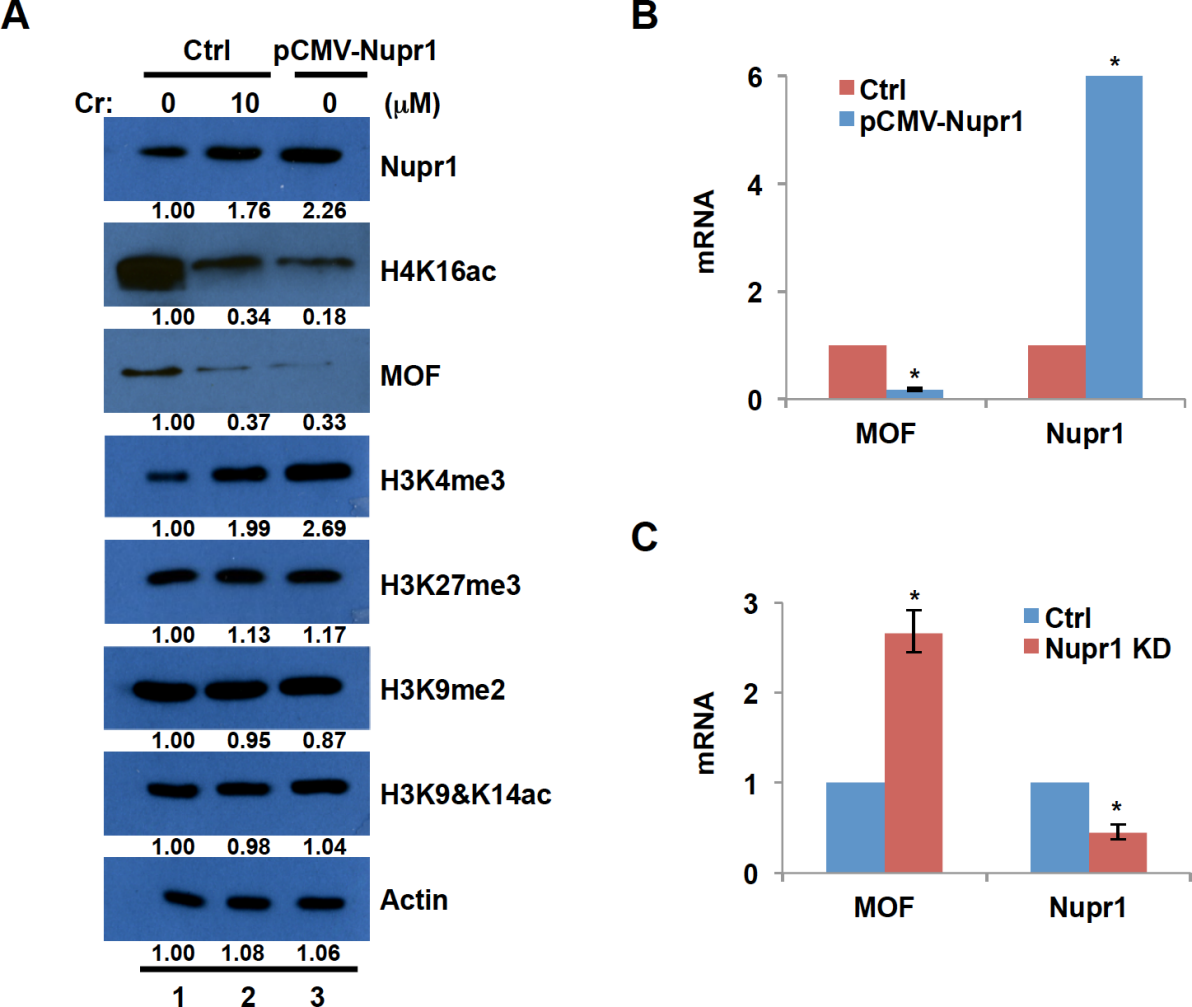
Overexpression of Nupr1 reduces both H4K16ac and histone acetyltransferase MOF and increases H3K4me3. (A) BEAS2B cells were transfected with Nupr1 expression vector or empty (Crtl) vector. The control cells were treated with or without Cr(VI) for 24 hours. The cell lysates were prepared with RIPA buffer, and run on a 14% SDS acrylamide gel. Western blot of Nupr1, H4K16ac, MOF, a number of other histone modifications and ß-actin was represented. Band intensities were measured by ImageJ software. (B and C) Total RNA was extracted either from BEAS2B cells that overexpress Nupr1 (B) or from Nupr1 knockdown cells (C). MOF and Nupr1 mRNA levels were measured by RT-qPCR and normalized to γ-tubulin. The transcription level was presented as fold change compared to the control group. The data shown are the mean ± S.D. from qPCRs performed in triplicate. *, *p*<0.01.

Next, we examined whether the DNA methylation and the histone modification status of Nupr1 promoter changed upon treatment with Cr(VI). Methylation-Specific PCR (MSP) was used to determine the DNA methylation status. DNA from the untreated cells produced a strong band with methylated primers, whereas DNA from the cells treated with 10 μM Cr(VI) for 24 hours generated much less amount of PCR products with methylated primers and more products with unmethylated primers as compared with untreated samples (Fig 2C, compare lanes 1 and 3, and 2 and 4 respectively). These results indicate that Nupr1 transcription was silenced by hypermethylation of CpG islands around the Nupr1 promoter in BEAS2B cells. We then applied ChIP assays to measure the level of histone H3K9 and H3K14 acetylations in the promoter region of Nupr1 before and after exposing cells to Cr(VI). Fig 3C shows that the level of H3K9 and H3K14 acetylations is significantly increased following the treatment. Thus, we conclude that induction of Nupr1 following Cr(VI) exposure is regulated by the changes in status of DNA methylation and histone acetylation in the promoter.

### Overexpression of Nupr1 reduces both H4K16 acetylation and MOF expression and increases H3K4me3

The loss of acetylation at histone H4K16 has been considered to be a ‘hallmark’ of cancers [29]. Histone acetyltransferase MOF forms a complex with MSL1 and MSL3 proteins and specifically acetylates H4K16 [39]. Interestingly, Gironella et al. reported that in HeLa cells MSL1-mediated H4K16ac is negatively regulated by overexpression of Nupr1 [22], suggesting that induction of Nupr1 by Cr(VI) could lead to a loss of H4K16ac. To test this hypothesis, we first determined how the level of H4K16ac is regulated by Cr(VI) exposure. BEAS2B cells that have been transfected with empty vector were treated with Cr(VI) and changes in H4K16ac were monitored by Western blot analysis. Exposure of cells to 10 μM Cr(VI) for 24 hours significantly reduced H4K16ac (Fig 3A, compare lanes 1 and 2). In addition, expression of MOF that acetylates H4K16 was also decreased when cells were exposed to Cr(VI) (Fig 3A), suggesting that Cr(VI) exposure leads to the loss of H4K16ac probably through downregulation of MOF expression or inhibition of MOF activity.

We have shown that Cr(VI) exposure up-regulates Nupr1 expression and induces the loss of H4K16ac. Is the Cr(VI)-induced reduction of H4K16ac resulting from the induction of Nupr1? To answer this question, we examined the level of H4K16ac upon overexpression of Nupr1. BEAS2B cells were transfected with Nupr1 plasmid or empty vector (control) and the levels of H4K16ac and MOF were measured by Western blot analysis. Fig 3A showed that Nupr1 protein level was increased by about 2.5-fold in cells transfected with pCMV-Nupr1 plasmid as compared with that in control cells (Ctrl) (compare lanes 1 and 3). Additionally, overexpression of Nupr1 led to decrease in the levels of both MOF and H4K16ac (lanes 1 and 3). The data imply that Cr(VI) exposure reduces H4K16ac probably through induction of Nupr1. We next examined whether other histone modifications are also changed by overexpression of Nupr1. Among active marks H3K4me3 and H3K9&K14ac, and repressive marks H3K27me3 and H3K9me2 that we tested, only the levels of H3K4me3 were apparently increased by Nupr1 overexpression or Cr(VI) exposure (Fig 3A). These results suggest that Cr(VI)-induced loss of H4K16ac and gain of H3K4me3 are mediated at least in part by the induction of Nupr1 expression.

MOF protein level was dramatically decreased by overexpression of Nupr1. To examine how Nupr1 modulates MOF expression, we determined whether the transcription level of MOF changes in response to Nupr1 overexpression or knockdown. BEAS2B cells were transiently transfected with empty vector or Nupr1-expressing plasmid and mRNA levels for Nupr1 and MOF were measured by RT-PCR. The mRNA level of Nupr1 was drastically increased in the cells transfected with Nupr1 plasmid, while the level of MOF was significantly reduced by overexpression of Nupr1 (Fig 3B). We next knocked down Nupr1 expression by siRNA and measured the transcription level of MOF in the cells transfected with control siRNA or siRNA specific for Nupr1. The mRNA level of MOF was increased by about 2.5 fold by the knockdown of Nupr1 (Fig 3C). We conclude that Nupr1 negatively regulates transcription of MOF.

### Knockdown of Nupr1 prevents BEAS2B cells from Cr(VI)-induced downregulation of H4K16ac and upregulation of H3K4me3

**Fig 4:**
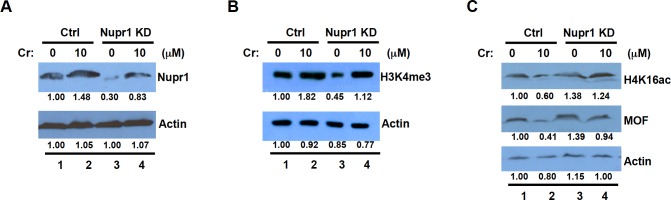
Knockdown of Nupr1 compromises Cr(VI)-induced downregulation of H4K16ac and upregulation of H3K4me3. BEAS2B cells transfected with control siRNA (Ctrl) or Nupr1 siRNA (Nupr1 KD) were grown under normal culture conditions for 48 h. After transfection, the cells were exposed to 5 or 10 μM Cr(VI) for 24 h. Cells were lysed with RIPA buffer and subjected to Western blot analysis with antibodies against Nupr1 (A), H3K4me3 (B), H4K16ac or MOF (C). Band intensities were measured by ImageJ software.

To determine the critical role for Nupr1 in Cr(VI)-induced changes in H4K16ac and H3K4me3, we knocked down Nupr1 expression by siRNA and examined how Cr(VI) exposure alters H4K16ac and H3K4me3 in the cells with siRNA for Nupr1. Nupr1 expression was decreased at least by 70% in the cells transfected with Nupr1 siRNA as compared with the control (Fig 4A, compare lanes 1 and 3). We next examined whether induction of Nupr1 by Cr(VI) is compromised in the cells transfected with siRNA for Nupr1. As expected, the amount of Nupr1 was increased following Cr(VI) exposure in the control cells (Fig 4A, compare lanes 1 and 2). Although the level of Nupr1 was also upregulated by Cr(VI) exposure in Nupr1 siRNA cells, the Nupr1 level was about 20% less than that in untreated control cells (Fig 4A, compare lanes 1 and 4). These data indicate that siRNA for Nupr1 efficiently reduces Nupr1 expression in Cr(VI)-exposed cells. We next investigated how the knockdown of Nupr1 affects Cr(VI)-induced changes in the levels of H3K4me3 and H4K16ac. The level of H3K4me3 was increased about 1.8-fold by Cr(VI) exposure in the control cells, while the level remained only about 80% in Nupr1 siRNA cells following Cr(VI) exposure (Fig 4B, compare lanes 2 and 4). Similarly, knockdown of Nupr1 greatly suppressed Cr(VI)-induced loss of H4K16ac and its modifying enzyme MOF (Fig 4C, lanes 2 and 4). The data support the idea that induction of Nupr1 is critical for the Cr(VI)-mediated loss of H4K16ac and gain of H3K4me3.

### Levels of H4K16ac are downregulated at several genomic loci by overexpression of Nupr1

**Fig 5:**
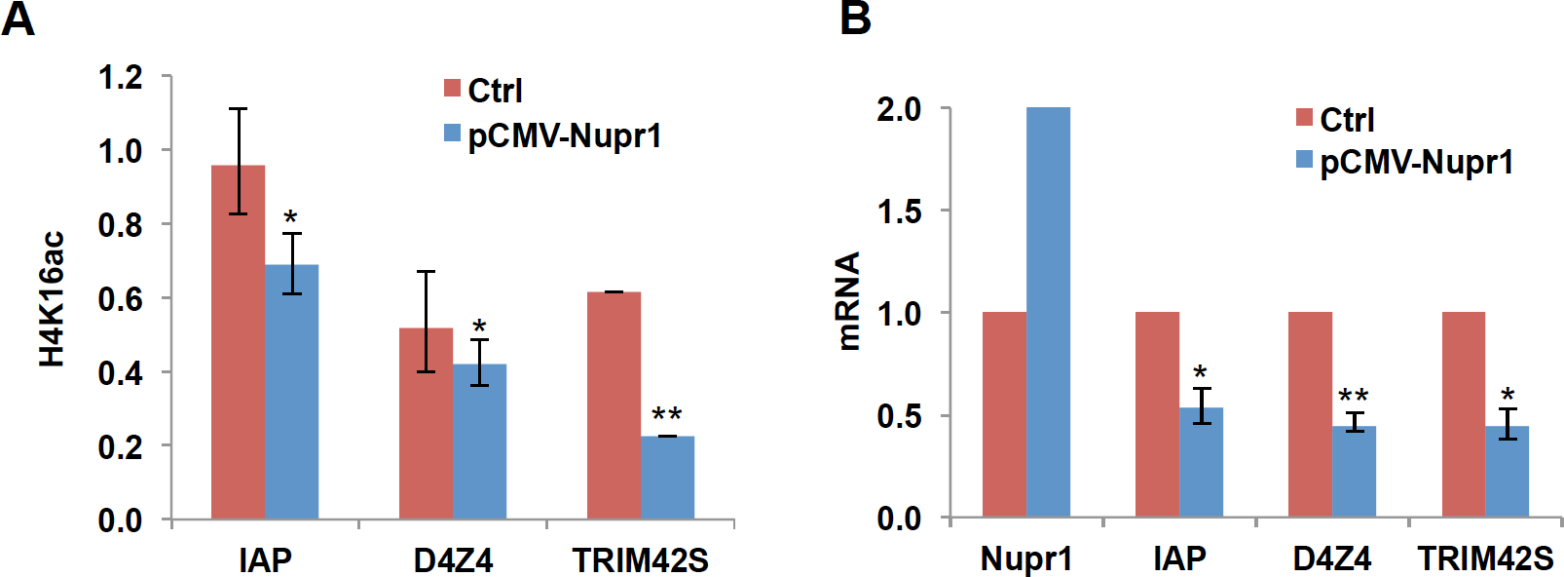
Reduction of H4K16ac induced by overexpression of Nupr1 correlates with transcriptional inactivation. (A) Levels of H4K16ac are downregulated at several genomic loci by overexpression of Nupr1. Mono- and dinucleosomes were prepared by MNase digestion from BEAS2B cells transfected with Nupr1 plasmid (pCMV-Nupr1) or empty vector (Ctrl) and subjected to ChIP assays with antibodies specific for H4K16ac. The enrichment of H4K16ac was measured by qPCR at several genomic loci including promoters of IAP (inhibitor of apoptosis), TRIM42S (tripartite motif-containing 42), and D4Z4 subtelomeric region. The data shown are presented as mean ± S.D. from qPCRs performed in triplicate. Relative-fold changes were calculated after normalization to input. *, *p*<0.05; **, *p*<0.01. (B) RT-qPCR measurements of transcripts in BEAS2B cells transfected with Nupr1 plasmid (pCMV-pur1) or empty vector (Ctrl). The amount of mRNA in control cells was set to 1. The data shown are the mean ± S.D. (error bars) from qPCRs performed in triplicate. *p<0.05; ** *p*<0.01.

We have shown that both overexpression of Nupr1 and Cr(VI) exposure dramatically reduce global levels of H4K16ac and increase H3K4me3 (Figs 1 and 3). H3K4me3 is a well-known transcriptional active mark localized in the active promoters. H4K16ac is enriched at the promoters of active genes and enhancers and seems to be associated with gene activity [40–43]. We performed ChIP and RT-qPCR to examine whether Nupr1-mediated changes in the levels of H4K16ac at selected gene loci correlate with transcription of corresponding genes. H4K16ac levels were reduced at promoters of TRIM42S and IAP genes and D4Z4 repeat array in the subtelomeric region in cells overexpressing Nupr1 (Fig 5A). The transcription levels of TRIM42S and IAP gene and D4Z4 region were accordingly downregulated (Fig 5B). These data suggest that Cr(VI) might inhibit gene expression by reducing H4K16ac at promoter and/or enhancer regions probably through upregulating Nupr1. The gain of a well-known transcriptional active mark H3K4me3 by induction of Nupr1 may contribute to Cr(VI)-induced activation of gene expression.

### Overexpression of Nupr1 induces cell transformation and knockdown of Nupr1 expression by small hairpin RNA prevents Cr(VI)-induced cell transformation

**Fig 6:**
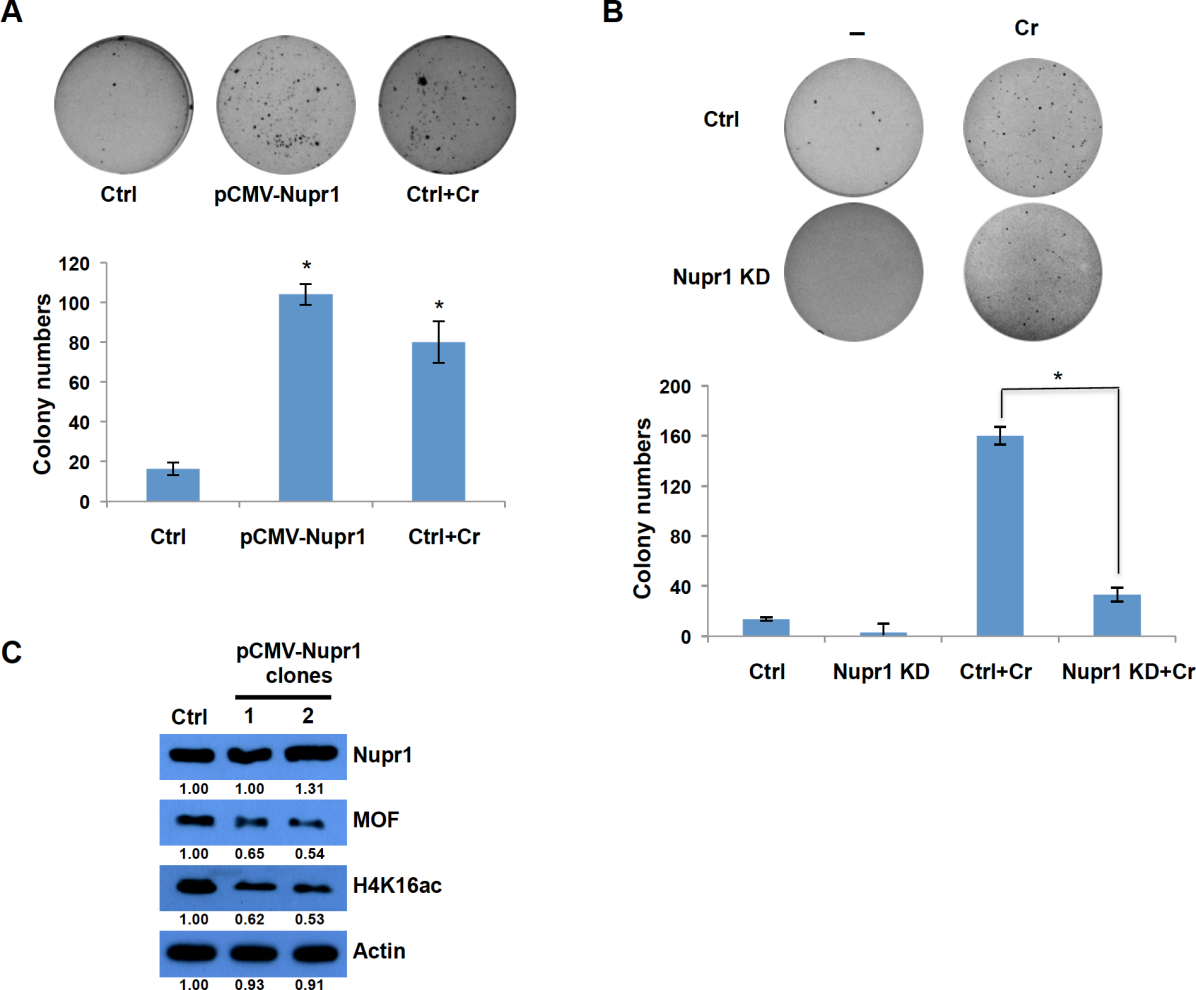
Role for Nupr1 in Cr(VI)-induced anchorage-independent growth of BEAS2B cells. (A) Overexpression of Nupr1 facilitates colony formation of BEAS2B cells on soft agar. BEAS2B cells were transiently transfected with empty vector (Ctrl) or Nupr1 expressing vector (pCMV-Nupr1). 48 h after the transfection, the cells were treated with or without 10 μM of Cr(VI) for 2 h, plated in 0.35% soft agar, and cultured for 3 weeks. (B) Knockdown of Nupr1 inhibits Cr(VI)-induced cell transformation. BEAS2B cells were transiently transfected with control siRNA (Ctrl) or Nupr1 siRNA (Nupr1 KD) for 48 hours and further grown in the presence or absence of 2 μM Cr(VI) for 72 h. The cells were then plated in 0.35% soft agar, and cultured for 3 weeks. The data shown are the mean ± S.D. from experiments performed in triplicate. *p<0.05. (C) Cell lysates were prepared either from two transformed cell clones derived from Nupr1 overexpression (clones 1 and 2) or from untreated BEAS2B cells (Ctrl) and subjected to Western blot analysis using the indicated antibodies. Band intensities were measured by ImageJ software.

Nupr1 is highly expressed in a number of cancer tissues including lung cancer [13, 18, 44, 45]. Induction of Nupr1 is required for cell transformation and cancer development in animal model. We have shown that Nupr1 expression is significantly induced by Cr(VI) exposure (Fig 1), indicating that induction of Nupr1 may play critical roles in Cr(VI)-mediated carcinogenesis. To investigate the role for Nupr1 in chromium-induced cell transformation, we utilized the BEAS2B cell transformation assays to investigate the effect that overexpression and knockdown of Nupr1, in the absence or presence of Cr(VI) exposure, has on the ability of these cells to acquire anchorage-independent growth. We exposed cells to 10 μM of Cr(VI) for 2 hours. BEAS2B cells that overexpress Nupr1 were capable of anchorage-independent cell growth (Fig 6A). Notably, whereas Cr(VI) exposure facilitated colony formation of BEAS2B cells transfected with control siRNA, knockdown of Nupr1 using siRNA for Nupr1 prevented the Cr(VI)-induced anchorage-independent growth (Fig 6B). The results indicate that induction of Nupr1 play critical roles in Cr(VI)-mediated cell transformation.

To address whether Nupr1 induces cell transformation through decreasing MOF and H4K16ac, we used transformed cell clones derived from Nupr1 overexpression (Fig 6A) to measure the levels of MOF and H4K16ac. The level of H4K16ac and MOF protein was apparently reduced in transformed clones as compared with untreated BEAS2B cells, whereas Nupr1 protein levels were similar in the control and transformed clones (Fig 6C). These data suggested that induction of Nupr1 might be transient and only required for initiation of cell transformation, whereas reduction of both MOF and H4K16ac was stable and required for both initiation and maintenance of cell transformation.

## Discussion

Cr(VI) is a well known human lung carcinogen. Epigenetic modification of gene expression has been considered to be a key element of the carcinogenic outcomes of exposure to Cr(VI), whereas the underlying mechanisms are not fully understood. Nupr1 is a stressor protein. Animal studies have demonstrated its critical role in the development of several cancers including lung cancer. However, the molecular function of Nupr1 has been elusive. In the presented study, we show that Nupr1 expression is induced by Cr(VI) exposure, leading to the loss H4K16ac, a ‘hallmark’ of cancer. Importantly, overexpression of Nupr1 induces anchorage-independent growth of lung epithelial BEAS2B cells, while knockdown of Nupr1 inhibits Cr(VI)-induced cell transformation. Thus, the induction of Nupr1 and subsequent loss of H4K16 acetylation might represent a novel mechanism for Cr(VI) carcinogenesis. To the best of our knowledge, this is the first study to investigate the role of Nupr1 in metal-induced carcinogenesis.

Nupr1 is induced by a variety of stressors and highly expressed in a number of human cancers. It has been unclear how Nupr1 expression is regulated. In the current study, we have demonstrated that Nupr1 mRNA and protein levels are upregulated by the treatment of the cells either with a histone deacetylase inhibitor (sodium butyrate) or by DNA methyltransferase inhibitor (5-azaC) (Fig 2A and 2B). We further showed that the level of DNA methylation in the Nupr1 promoter was shifted from hyper- to hypo-methylation, while the levels of active marks H3K9,14 acetylation were increased following Cr(VI) exposure (Fig 2C and 2D). These data imply that Nupr1 transcription is induced by Cr(VI) through epigenetic mechanisms. Cr(VI) might target histone and DNA modifying enzymes to change the epigenetic status of Nupr1 promoter. For example, the demethylase JARID1 specific for H3K4me3 contains an oxidation sensitive Fe required for the enzyme activity and the activity of JARID1 could be inactivated by Cr(VI) oxidation of Fe^+2^ to Fe^+3^. This would increase the active mark H3K4me3 globally and in the promoter of Nupr1 resulting in its activation. This is supported by our observation of a specific increase in global levels of H3K4me3 when BEAS2B or human lung A549 cells are exposed to Cr(VI) (Fig 3A). In the future, we will examine whether the activity of JARID1 and other histone and DNA modifying enzyme(s) is disrupted following Cr(VI) exposure. It would also be interesting to define if antioxidants such as vitamin C, E, EGCG, catalase and superoxide dismutase (SOD) suppress the ability of Cr(VI) to inhibit activity of chromatin modifying enzyme(s) such as JARID1.

Elegant work from Alvaro Puga’s group has demonstrated that Cr(VI) treatment preferentially opens chromatin around AP1 binding sites [46]. Moreover, transcription factor ChIP-seq results from ENCODE summarized in UCSC Genome Browser showed the binding of AP1 factors such as JUND and FOS around the transcription start site (TSS) and -2 kb upstream of the Nupr1 gene (not shown). Thus, AP1 transcription factors might be responsible for Cr(VI)-induced activation of Nupr1 expression. We previously reported that the AP1 inhibitor protein JDP2 binds to AP1 site of c-Jun gene to suppress its expression by recruiting HDAC3 [47], while c-Jun expression is induced following retinoic acid exposure by the release of JDP2 from the AP1 site. Cr(VI) has been shown to prevent the expression of some genes in cells by crosslinking histone deacetylase 1 to the promoters [8]. The presence of this deacetylase, which removes acetyl groups from lysines in histone tails, keeps the nucleosome condensed, thereby preventing transcription factors from binding and activating gene expression [8]. This raised interesting possibility that the loss of expression of a Nupr1 suppressor, e.g., JDP2, by HDAC crosslinking could activate Nupr1.

Overexpression of Nupr1 increased global levels of H3Kme3 and H4K16ac (Fig 3A). H3K4me is enriched in the active promoters and is a well-known transcription active mark. H4K16ac is localized at enhancers and promoters of active genes. Moreover, H4K16ac is involved in chromatin decondensation. Thus, transcriptional regulation by Nupr1-mediated gain of H3K4me3 and loss of H4K46ac might have impact on biological processes following Cr(VI) exposure. In agreement with this proposal, the ChIP results showed reduction of H4K16ac at several genomic loci, which correlates with suppression of gene expression (Fig 5). Interestingly, we also found the increase of H4K16ac by overexpression of Nupr1 at several genomic loci without increase in transcription of corresponding genes (data not shown). These data indicate that reduction of H4K16ac alone is sufficient for inhibition of gene expression, whereas upregulation of H4K16ac requires other mechanisms involved in gene activation. At least in yeast, it seems that the presence of H4K16ac may poise many genes toward to become rapidly activated but by itself is not sufficient to stimulate transcription [48]. Nupr1 binds to MSL1, a component of MOF-MSL complex, thereby inhibiting MOF activity [22]. In addition, Nupr1 was found to interact with transcription factors such as p53, p300, and SMAD etc. and regulates transcription of its downstream genes [49]. Thus, Cr(VI) exposure might interfere with interaction between Nupr1 and its partners, leading to biological effects.

Nupr1 is highly expressed in a number of cancer tissues including lung cancer [13, 18, 44, 45]. Induction of Nupr1 is required for cell transformation and cancer development in animal models. When mouse embryo fibroblasts (MEFs) from wild-type and Nupr1-null mice were transformed with rasV12 mutant and E1A oncogene, Nupr1-/- MEFs could not form colonies in soft agar and no tumor was observed with transformed Nupr1-/- MEFs following subcutaneaous or intraperitoneal injections [50]. In a mouse model of pancreatic cancer with constitutively expressed oncogenic Kras(G12D), loss of Nupr1 protected from the development of pancreatic intraepithelial neoplasias (PanINs) [32]. Moreover, knockdown of Nupr1 by lentivirus-mediated RNAi significantly inhibited colony formation of human non-small cell lung cancer H1299 *in vitro* and tumor growth *in vivo* by tail vein injection of shRNA against Nupr1 [18]. Moreover, both Cr(VI) exposure and Nupr1 overexpression led to the loss of H4K16ac and gain of H3K4me3. The global loss of H4K16ac is considered as a common hallmark of human cancers [29]. Using immunodetection, high-performance capillary electrophoresis and mass spectrometry, Fraga et al. analyzed the levels of H4K16ac in 36 primary tumors, including lymphoma and lung cancer, and 25 cancer cell lines corresponding to human tumors. They found global reduction in H4K16ac and this loss was associated with the hypomethylation of DNA repetitive sequences,a characteristic of cancer cells [29]. Other groups also reported the hypoacetylation of H4K16 in lung cancer cell lines and in 70% of 100 lung cancer tissues, and inverse correlation of H4K16ac with recurrence of lung cancer [27, 28, 51]. Importantly, our data have shown that overexpression of Nupr1 induces anchorage-independent growth, while knockdown of Nupr1 inhibits Cr(VI)-induced cell transformation (Fig 6). Therefore, induction of Nupr1 expression might play important roles in chromium-induced cancer development.

In summary, in this study we have found that we Cr(VI) significantly induces a small stressor protein Nupr1, which results in perturbation of epigenetic profiles and transcription and leads to transformation of lung epithelial cells. Our work highlights how cellular stress responses to Cr(VI) exposure were handled via Nupr1-dependent regulation of the epigenetic program and and its potential impact on Cr(VI) carcinogenesis.
